# Cannabidiol Regulates the Expression of Keratinocyte Proteins Involved in the Inflammation Process through Transcriptional Regulation

**DOI:** 10.3390/cells8080827

**Published:** 2019-08-03

**Authors:** Anna Jastrząb, Agnieszka Gęgotek, Elżbieta Skrzydlewska

**Affiliations:** Department of Analytical Chemistry, Medical University of Bialystok, 15-089 Bialystok, Poland

**Keywords:** cannabidiol, UV radiation, keratinocytes, antioxidants, inflammation, intracellular signaling, Nrf2, NFκB

## Abstract

Cannabidiol (CBD), a natural phytocannabinoid without psychoactive effect, is a well-known anti-inflammatory and antioxidant compound. The possibility of its use in cytoprotection of cells from harmful factors, including ultraviolet (UV) radiation, is an area of ongoing investigation. Therefore, the aim of this study was to evaluate the effect of CBD on the regulatory mechanisms associated with the redox balance and inflammation in keratinocytes irradiated with UVA [30 J/cm^2^] and UVB [60 mJ/cm^2^]. Spectrophotometric results show that CBD significantly enhances the activity of antioxidant enzymes such as superoxide dismutase and thioredoxin reductase in UV irradiated keratinocytes. Furthermore, despite decreased glutathione peroxidase and reductase activities, CBD prevents lipid peroxidation, which was observed as a decreased level of 4-HNE and 15d-PGJ_2_ (measured using GC/MS and LC/MS). Moreover, Western blot analysis of protein levels shows that, under stress conditions, CBD influences interactions of transcription factors Nrf2- NFκB by inhibiting the NFκB pathway, increasing the expression of Nrf2 activators and stimulating the transcription activity of Nrf2. In conclusion, the antioxidant activity of CBD through Nrf2 activation as well as its anti-inflammatory properties as an inhibitor of NFκB should be considered during design of new protective treatments for the skin.

## 1. Introduction

It is believed that various disorders of human skin are the result of oxidative stress occurring in skin cells. Therefore, maintaining the redox balance is very important, especially because the skin remains in constant contact with the environment, plays an important role in the body’s response to environmental factors, and also mediates the transmission of environmental signals to the organism [1]. Exposure to solar UV radiation is a causative factor in acute skin photodamage, chronic photoaging, and photocarcinogenesis [2,3]. UVA and UVB radiation have different biological effects on skin cells, but the common feature is to cause a redox imbalance with a shift towards oxidation. This is a result of increased reactive oxygen species (ROS) generation, which under physiological conditions are involved in cell signaling, but in the case of overproduction can also lead to cell damage. Cell damage is favored in the setting of UV radiation due to the reduction of effective endogenous antioxidant protection [4].

Oxidative stress caused by both endogenous and environmental factors reflects an imbalance between the number of oxidants produced in skin cells and the amount of antioxidant gene products (superoxide dismutase [SOD], catalase [CAT], glutathione [GSH], peroxidase [Px], etc.) maintaining the redox balance [5]. From this point of view, the expression of Nrf2, a transcription factor ubiquitously expressed in all tissues, including skin, which modulates the levels of cytoprotective protein expression, seems very important. Nrf2 protein levels and its activity are tightly regulated [6]. Activation is significantly related to the oxidative modification of the cysteine reactant Keap1, its cytosol inhibitor, which is responsible for targeting Nrf2 to ubiquitination and proteasomal degradation [7]. In contrast, the activation mechanisms of Nrf2 involve kinase signaling pathways, with Nrf2 phosphorylation being ultimately responsible for its translocation into the cell nucleus [8]. Natural compounds, such as sulforaphane and its derivatives, as well as various drugs, also play a role as activators or inhibitors of Nrf2 [9,10].

**Figure 1:**
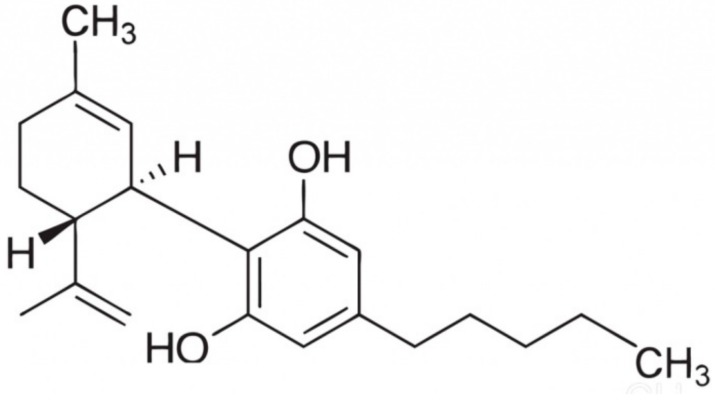
Cannabidiol chemical structure.

One of the natural compounds that can support the skin’s antioxidant system is the main component of the Cannabis sativa extract, cannabidiol (CBD). CBD is a phytocannabinoid, which has no psychoactive effect, but aroused interest due to the therapeutic potential for many disease states that have been studied in animal models [11,12,13,14]. It has antioxidant, anti-inflammatory, anxiolytic, antitumor and anti-cancer properties [15,16]. Due to the chemical structure (Figure 1), CBD, like other antioxidants, interferes with the free radical chain reactions mainly in the propagation and the final phase, capturing free radicals or transforming them into less reactive forms [17]. In addition, CBD leads to a decrease in oxidative conditions in cells, which is associated with chelation of transition metals and reduction of prooxidative enzymes, which leads to prevention of ROS [18]. Notwithstanding the reduction of levels of oxidants, CBD modifies the redox balance also by changing the level/activity of antioxidants [19].

CBD, like endocannabinoids, acts not only directly, but also indirectly through receptors. By activating A2A adenosine receptors, it inhibits the NFκB inflammatory pathway and reduces the level of proinflammatory cytokines, including TNFα [20]. This phytocannabinoid has a low affinity for cannabinoid receptor 1 (CB1) and behaves like an incompetent antagonist; therefore, it can increase stimulation of the cannabinoid system. It inhibits the anandamide transport and its degradation of by fatty acid amide hydrolase [21]. In addition, CBD exhibits weak inverse agonism towards the cannabinoid receptor 2 (CB2). It can act as an “indirect” CB1/CB2 agonist by weak inhibition of anandamide enzymatic hydrolysis [22]. CBD may also have effects at the level of other endocannabinoids, including 2-arachidonylglicerol (2-AG) [23]. Moreover, it modulates the activity of G protein-coupled receptors (GPCRs) that are activated by endocannabinoids and related compounds [24].

The broad action of CBD does not remain indifferent to the genetic material. It has been found that CBD by inhibition of the DNA repair enzyme PARP-1 can induce cytotoxicity [25] and acts destructively in relation of DNA of cancer-transformed cells therefore CBD has been suggested as a promising antitumor factor [26]. Moreover, anti-inflammatory and modulatory effects of CBD promote the attenuation of various autoimmune conditions in animal models, including skin diseases such as psoriasis [27]. In addition, by affecting skin cells, it inhibits the proliferation and differentiation of human keratinocytes via CB1 and CB2 independent epigenetic mechanisms [28].

Therefore, the aim of this study was to evaluate the effect of CBD on regulatory mechanisms related to redox balance and inflammation in keratinocytes after UVA and UVB irradiation.

## 2. Materials and Methods

### 2.1. Cell Culture and Treatment

Human keratinocytes (CDD 1102 KERTr) were obtained from American Type Culture Collection (ATCC, Virginia, USA). Keratinocytes were cultured in Keratinocyte Serum-Free Medium (Gibco, Grand Island, NY) that contained fetal bovine serum (10%), epidermal growth factor (EGF 1–53; 5 µg/L), 50 U/mL penicillin and 50 μg/mL streptomycin. Cells were cultured in a humidified atmosphere with 5% CO_2_ at 37 °C. When the cells (passage 9–11) reached 70% confluency, they were washed with warm PBS (37 °C). Cells were exposed to UV radiation in cold PBS (4 °C) to avoid heat stress and oxidation of the medium components. The exposure dose that corresponded to 70% cell viability measured by the MTT assay was used [29]. The cells were irradiated on ice at a distance of 15 cm from the assembly of 6 lamps (Bio-Link Crosslinker BLX 312/365; Vilber Lourmat, Germany), 6W each, which corresponds to 4.2 mW/cm^2^ and 4.08 mW/cm^2^, respectively for UVA (365 nm) and UVB (312 nm). Total radiation doses were 30 J/cm^2^ UVA and 60 mJ/cm^2^ UVB. After radiation, cells were incubated for 24 h under standard conditions without rinsing; control cells that did not receive irradiation were incubated in parallel.

To examine the effect of CBD on keratinocytes all cell groups, control cells and cells after UVA and UVB irradiation were cultured in medium containing 1 µM CBD (THC-Pharm, Frankfurt, Germany). After 24 h incubation, all cells were washed with PBS, collected by scraping into cold PBS and centrifuged. Cells were then resuspended in PBS and subjected to sonication. Total protein content in cell lysate was measured using a Bradford assay [30].

### 2.2. Cell Viability

To examine the effect of CBD on UV irradiated keratinocytes, cells were incubated after irradiation for 24 h under standard conditions in medium containing 0, 0.1, 0.5, 1, 2, 4, 10, 25, 50, 100 nmoles/mL CBD in ethanol. The MTT assay was used to examine the effect of CBD concentration on keratinocyte viability compared to control cells [29].

### 2.3. Determination of Superoxide Anion Generation

The generation of superoxide anions was detected using an electron spin resonance (ESR) spectrometer e-scan (Noxygen GmbH/Bruker Biospin GmbH, Berlin, Germany), where selective interaction of the spin probes CMH (1-hydroxy-3-methoxy-carbonyl-2,2,5,5-tetrame-thylpyrrolidine, 200 µM) with ROS formed a stable nitroxide CM-radical with a half-life of 4 h. Thus, ROS formation was measured by assessing the kinetics of nitroxide accumulation based on the electron spin resonance amplitude of the low field component of ESR spectra. The rate of superoxide radical formation was determined by measuring superoxide dismutase (SOD)-inhibited nitroxide generation [31].

### 2.4. Determination of Antioxidant Enzyme Activity

SOD (Cu/Zn–SOD, EC.1.15.1.1) activity was determined according to the method of Misra and Fridovich [32] as modified by Sykes and co-authors [33], which measures the activity of cytosolic SOD. One unit of SOD was defined as the amount of the enzyme that inhibits epinephrine oxidation to adrenochrome by 50%. Enzyme specific activity was expressed in units per milligram of protein.

Glutathione peroxidase (GSH-Px, EC.1.11.1.6) activity was assessed spectrophotometrically using the method of Paglia and Valentine [34]. GSH-Px activity was assayed by measuring the conversion of NADPH to NADP^+^. One unit of GSH-Px activity was defined as the amount of enzyme catalyzing the oxidation of 1 µmol NADPH/min at 25 °C and pH 7.4. Enzyme specific activity was expressed in units per mg of protein.

Glutathione reductase (GSSG-R, EC.1.6.4.2) activity was measured according to the method of Mize and Longdon [35] by monitoring the oxidation of NADPH at 340 nm. One unit of GSSG-R oxidized 1 mmol of NADPH/min at 25 °C and pH 7.4. Enzyme specific activity was expressed in milliunits per milligram of protein.

The thioredoxin reductase (TrxR, EC. 1.8.1.9) activity was measured using a commercially available kit (Sigma-Aldrich, St. Louis, MO, USA). The principle on which this kit is based is the NADPH-mediated reduction of 5,5′-dithiobis(2-nitrobenzoic) acid (DTNB) to 5-thio-2-nitrobenzoic acid (TNB), which produces a strong yellow color that is measured at 412 nm [36]. Enzyme activity was expressed in units per milligram of protein.

### 2.5. Determination of Non-Enzymatic Antioxidant Level

Glutathione was quantified using the capillary electrophoresis (CE) method of Maeso and co-authors [37]. Samples were sonicated in Eppendorf tubes with 2 mL of a mixture containing ACN/H_2_O (62.5:37.5, *v*/*v*) and centrifuged at 29,620× *g* for 10 min. The supernatant was immediately subjected to CE. The separation was performed on a capillary with 47 cm total length (40 cm effective length) and 50 m i.d. and was operated at 27 kV with UV detection at 200 ± 10 nm. The GSH concentration was determined using a calibration curve range: 1–120 nmol/L (r^2^ = 0.9985) and the level of GSH was expressed as nanomoles per milligram of protein.

Thioredoxin level was quantified using the ELISA method [38]. Prepared standards and cell lysates were loaded into ELISA plate wells (Nunc Immuno Maxisorp, Thermo Scientific, Waltham, MA, USA) and incubated at 4 °C overnight with anti-thioredoxin primary antibody (Abcam, Cambridge, MA, USA). After washing, the plates were incubated for 30 min with peroxidase blocking solution (3% H_2_O_2_, 3% fat free dry milk in PBS) at RT. The goat anti-rabbit secondary antibody solution (Dako, Carpinteria, CA, USA) was then added to each well, and the plates were incubated for 1 h at RT. Chromogen substrate solution (0.1 mg/mL TMB, 0.012% H_2_O_2_) was added to each well, and the plates were incubated for 40 min at RT. The reaction was stopped by addition of 2M sulfuric acid. Absorption was read at 450 nm with the reference filter set to 620 nm. Level of thioredoxin was expressed as the micrograms per milligram of protein.

### 2.6. Determination of Lipid Peroxidation Product

Lipid peroxidation was estimated by measuring the level of 4-hydroxynonenal (4-HNE). Aldehyde was measured by GC/MSMS (Agilent Technologies, Palo Alto, CA, USA), as the *O*-PFB-oxime-TMS derivative, using a modified method of Liu and co-authors [39]. Benzaldehyde-D_6_ was added to the cell lysates as an internal standard, and aldehydes were derivatized by the addition of *O*-(2,3,4,5,6-pentafluoro-benzyl) hydroxyamine hydrochloride (0.05 M in PIPES buffer, 200 µL) and incubating for 60 min at RT. After incubation, samples were deproteinized by the addition of 1 mL of methanol and *O*-PFB-oxime aldehyde derivative was extracted by the addition of 2 mL of hexane. The top hexane layer was transferred into borosilicate tubes, and evaporated under a stream of argon gas, followed by the addition of *N,O*-bis(trimethylsilyl)trifluoroacetamide in 1% trimethylchlorosilane. A 1 µL aliquot was injected on the column. Derivatized aldehyde was analyzed using a 7890A GC–7000 quadruple MS/MS (Agilent Technologies, Palo Alto, CA) equipped with a HP-5ms capillary column (0.25 mm internal diameter, 0.25 µm film thickness, 30 m length). Derivatized aldehyde was detected in selected ion monitoring (SIM) mode. The ions used were: *m*/*z* 333.0 and 181.0 for 4-HNE-PFB-TMS and *m*/*z* 307.0 for IS derivative. Level of 4-HNE was expressed as the nanomoles per milligram of protein.

### 2.7. Determination Of Anti-Inflammatory Eicosanoid

15-deoxy-Δ12,14-prostaglandin J2 (15d-PGJ2) was estimated using ultra-performing liquid chromatography tandem mass spectrometry (LCMS 8060, Shimadzu, Kyoto, Japan) [40]. PGF2α-d4 was used as an internal standard for quantification. Lipid mediators were extracted using SPE and analyzed in negative-ion mode (MRM). The precursor to the product ion transition was *m*/*z* 315.2→271.2 for 15d-PGJ2. The level of 15d-PGJ2 was expressed as picomoles per milligram of protein.

### 2.8. Determination of Protein Expression

Western blot analysis of cell protein was performed according to Eissa and Seada [41]. Whole cell lysates or membrane fractions were mixed with sample loading buffer (Laemmli buffer containing 5% 2-mercaptoethanol), heated at 95 °C for 10 min, and separated using 10% Tris-Glycine SDS-PAGE. Separated proteins in the gels were electrophoretically transferred to nitrocellulose membrane. The membrane was blocked with 5% skim milk in TBS-T buffer (Tris buffered saline with 5% Tween-20) for 1 h. Primary monoclonal antibodies raised against phospho-Nrf2 (pSer40), HO-1, Keap1, WTX, DPP3, CBP, TNFα, NFκB (p52), IKKα, IKKβ, phosphor-IκB (pSer36), p38, phospho-MAPK (phospho-p44/42), phospho-ASK-1 (pThr845) PGAM5, NLRP3, β-actin (Sigma-Aldrich), and Bach1, KAP1, p21, p62, Ref-1, NFκB (p65) (Santa Cruz Biotechnology, Santa Cruz, CA, USA) were used at a concentration of 1:1000. Alkaline phosphatase-labeled secondary antibodies against mouse, rabbit or goat were used at a concentration of 1:1000 (Sigma-Aldrich, St. Louis, MO, USA). Protein bands were visualized using the BCIP/NBT Liquid substrate system (Sigma-Aldrich) and were quantitated using the Versa Doc System and Quantity One software (Bio-Rad Laboratories Inc., CA, USA). Level of proteins was expressed as the percentage of the value determined from the control cells

### 2.9. Determination of Keap1 Structure

Human Keap1 (Sino Biological, cat. 11981-H20B) was dissolved in 25 mM AMBIC and divided into portions containing 30 µg of protein. One sample was incubated for 1 h at 4 °C with 10 µM CBD, while an untreated sample was used as a control. Each sample was digested with trypsin (1:50) for 4 h at 37 °C. Digestion was stopped by addition of 10% formic acid (FA) to a final concentration 0.1%. Samples were dried, resuspended in 50 µl ACN + 0.1% FA, and separated using an Ultimate 3000 (Dionex, Idstein, Germany) onto a 150 mm × 75 mm PepMap RSLC capillary analytical C18 column with 2 μm particle size (Dionex, LC Packings, Idstein, Germany). The peptides eluted from the column were analyzed using a Q Exactive HF mass spectrometer with an electrospray ionization source (ESI) (Thermo Fisher Scientific, Bremen, Germany) as described previously [42]. Data were acquired with the Xcalibur software (Thermo Fisher Scientific, Bremen, Germany).

### 2.10. Determination of Protein Localization

Cells were seeded in BD Falcon™ 96-well clear bottom black tissue culture plates optimized for imaging applications at 10,000 cells per well. After treatment with UV radiation and/or a 24 h incubation with CBD, cells were fixed with 3.7% formaldehyde solution for 10 min and permeabilized with 0.1% Triton X-100 solution for 5 min. Next, the cells were washed twice with PBS, and non-specific binding was blocked by adding a 3% FBS solution for 30 min. After that time, the cells were incubated with an anti-Nrf2 rabbit polyclonal antibody or an anti-NFκB mouse polyclonal antibody (Sigma-Aldrich; 1:1000) for 1 h at RT, washed three times with PBS and incubated with fluorescent (FITC) anti-rabbit or anti-mouse secondary antibody (BD Pharmingen, San Diego, CA) for 60 min in the dark. After washing, nuclei were stained with Hoechst 33342 (2 µg/mL) and analyzed using microscopy imaging. Images of FITC-labeled cells were acquired using a 488/10 excitation laser and a 515LP emission laser.

### 2.11. Statistical Analysis

Data were analyzed using standard statistical analyses, including ANOVA, and the results are expressed as the mean ± standard deviation (SD) for *n* = 5. P-values less than 0.05 were considered significant.

## 3. Results

**Figure 2:**
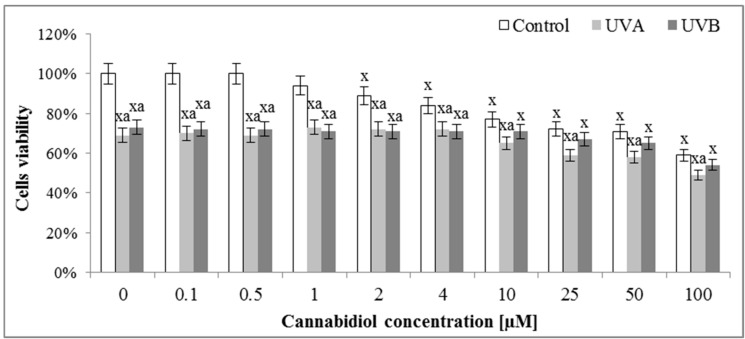
The influence of cannabidiol (CBD) on viability of keratinocytes exposed to UVA (30 J/cm^2^) and UVB (60 mJ/cm^2^) irradiation measured by MTT test. Mean values ± SD of five independent experiments are presented: ^x^ statistically significant differences vs. group without radiation or chemical treatment, *p* < 0.05; ^a^ statistically significant differences vs. UVA or UVB group, *p* < 0.05.

The assessment of the effect of different concentrations of CBD on the survival of keratinocytes by means of the MTT test indicated that concentrations of up to 1 μM CBD did not affect the survival of control keratinocytes (Figure 2). Higher concentrations caused a gradual decrease in cell survival, so that at a concentration of 10 μM it reached values of about 75% of control cells viability. In the case of cells irradiated with UVA radiation, both in the absence of CBD and in the presence of CBD up to 4 μM, the survival rate was maintained at 70%, and at higher concentrations was further reduced. In contrast, keratinocytes irradiated with UVB radiation were characterized by approximately 70% survival from 0 to 10 μM CBD and at higher CBD concentrations, the viability was somewhat reduced.

**Figure 3:**
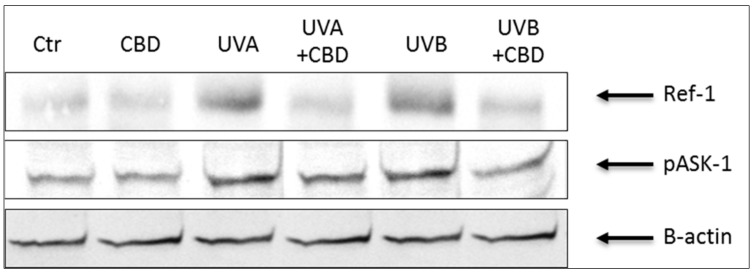
The influence of cannabidiol (CBD) on Ref-1 and phospho-ASK-1 (pThr845) levels in keratinocytes exposed to UVA (30 J/cm^2^) and UVB (60 mJ/cm^2^) irradiation.

Results showed that keratinocytes irradiated with UVA or UVB were characterized by an altered redox balance associated with a more than 2-fold increase in superoxide anion production and a significant decrease in Cu, Zn-SOD and TrxR activity, especially after UVB irradiation (Table 1). Moreover, UV radiation enhanced the cytoprotective factors phospho-ASK-1 and Ref-1 expression by about 2- and 3-fold (Table 1, Figure 3), respectively. These changes were accompanied by a significant reduction in the level of non-enzymatic antioxidant parameters, such as GSH and Trx. As a consequence, despite the increase in the activity of enzymes involved in the GSH metabolism, oxidative metabolism of phospholipids increased 2-3 times after UVA or UVB exposure, which was manifested by an increased level of the lipid peroxidation product, 4-HNE. Treating keratinocytes with CBD significantly reduced the production of ROS, especially after UVA irradiation. In addition, CBD increased the antioxidant capacity manifested by significantly increased activity of Cu, Zn-SOD and TrxR and the level of Trx. On the other hand, the UV-increased Ref-1 and phospho-ASK-1 expression was partially restored following CBD treatment. However, after the use of CBD, the GSH-Px and GSSG-R activity was reduced, as well as GSH levels in control or irradiated cells. The consequence of the observed CBD-induced changes in redox parameters was the reduction in the intensity of lipid peroxidation, which was seen as a significant decrease in the level of 4-HNE, especially in keratinocytes treated with CBD after UVA irradiation.

**Figure 4:**
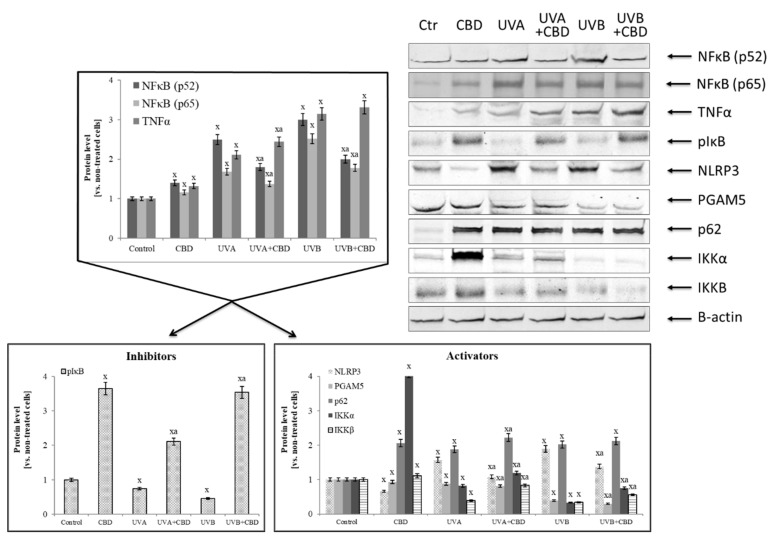
The influence of cannabidiol (CBD) on NFκB [p52] and NFκB [p65] transcriptional activity and its activators and inhibitors expression in keratinocytes exposed to UVA (30 J/cm^2^) and UVB (60 mJ/cm^2^) irradiation. Mean values ± SD of five independent experiments are presented. ^x^ statistically significant differences vs. control group, *p* < 0.05; ^a^ statistically significant differences vs. group without cannabidiol, *p* < 0.05.

Irradiation of keratinocytes with UV not only changed the activity and level of antioxidants, but also the expression and transcriptional activity of Nrf2 and NFκB, two transcription factors whose biological activity depends on each other and which participate in the modulation of redox imbalance and inflammation. UVA and UVB radiation increased the expression of NFκB (p52 and p65), while CBD treatment only partially, but significantly reduced this increase (Figure 4). However, keratinocytes treated with CBD alone were characterized by increased expression of NFκB. In addition, CBD used alone or after irradiation enhanced the expression of TNFα, an NFκB transcriptional activity product. Analyzing the NFκB activators and inhibitors, it can be concluded that the expression of the phosphorylated form of the basic NFκB inhibitor, IκB, was lowered by UV radiation and was significantly increased by CBD, particularly after UVB irradiation. In contrast, in the case of NFκB activators, CBD acted in a different way. It inhibited the expression of factors associated with the activation of inflammation, such as NLRP3 and PGAM5, and simultaneously activated IKK components, especially IKKα, whose expression was lowered by UV radiation as well as the protein p62 that is a direct linking element of the NFκB and Nrf2 pathways.

**Figure 5:**
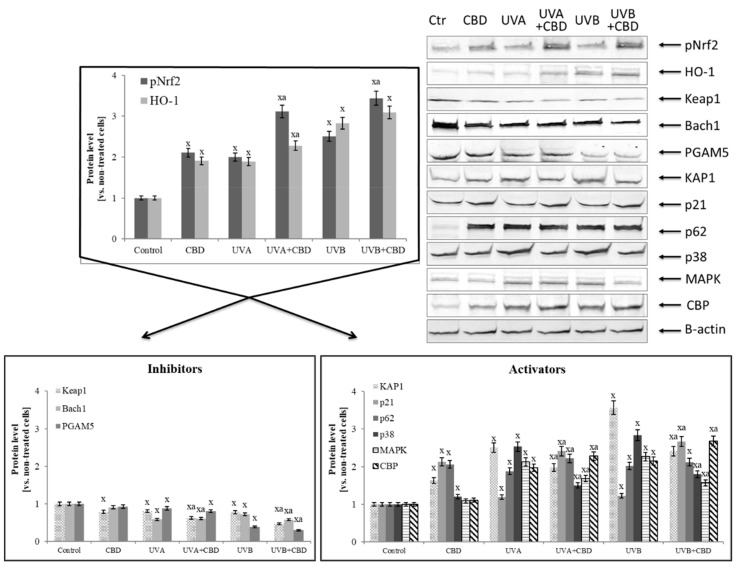
Effect of cannabidiol (CBD) on Nrf2 transcriptional activity and the level of its activators and inhibitors in keratinocytes exposed to UVA (30 J/cm^2^) and UVB (60 mJ/cm^2^) irradiation. Mean values ± SD of five independent experiments are presented: ^x^ statistically significant differences vs. control group, *p* < 0.05; ^a^ statistically significant differences vs. group without cannabidiol, *p* < 0.05.

Increased expression of NFκB (p52 and p65) by CBD promoted the increased expression of Nrf2 responsible for the transcription of cytoprotective proteins, which is confirmed by the increased biosynthesis of HO-1 and Trx and TrxR (Table 1, Figure 5). At the same time, the level of basic inhibitors Nrf2, cytosolic Keap1 and the nuclear Bach1 was decreased, as well as the level of PGAM5 that participate in inflammatory processes. Moreover, CBD stimulated the expression of Nrf2 activators (such as KAP1, p21, p62) in control keratinocytes two-fold. In contrast, in cells irradiated with UV, CBD only slightly increased the expression of p21 and p62 proteins.

Analyzing the decrease in the expression of the basic Nrf2 cytosolic inhibitor Keap1, it can be concluded that it was associated with a disruption of both inhibitors and activators of this factor (Table 2). In the case of the activators, the most unambiguous changes were observed in the DPP3 level, the expression of which was increased two-fold in all groups after the use of CBD. On the other hand, CBD significantly reduced the expression of another Keap1 activator, protein WTX. In the case of Keap1 inhibitors, CBD strongly increased 15d-PGJ_2_ level in the control cells, but after exposure to UV radiation the CBD effect was observed as a reduction in the level of this eicosanoid.

**Figure 6:**
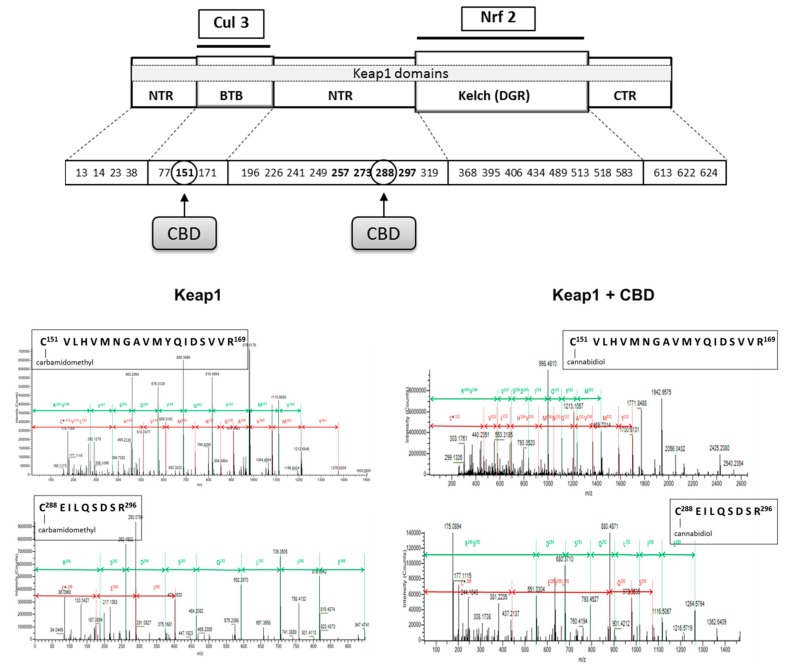
The localization of cysteine residues within Keap1 domains that form adducts with cannabidiol (CBD). Bolded cysteine residues are the most sensitive for oxidation.

In contrast, CBD in all cases reduced the level of inhibitors such as 4-HNE and PGAM5. Additionally, CBD influenced Keap1 activity by adduct formation on cysteine-288, which is responsible for Keap1 conformation and its interaction with Nrf2, as well as on cysteine-151 that is present in a Keap1 region responsible for Cul3 binding and indirectly contributes to Nrf2 ubiquitination (Figure 6).

**Figure 7:**
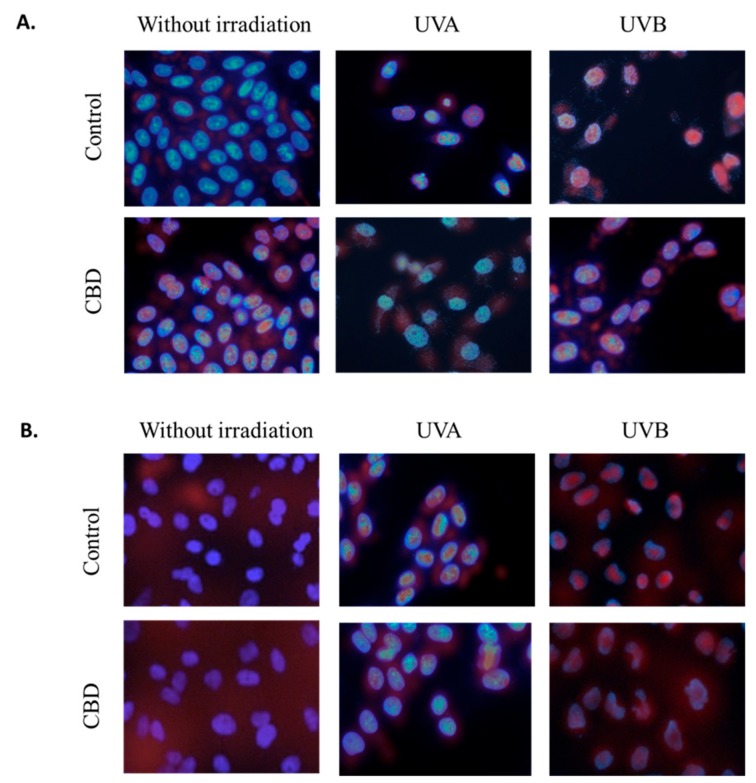
The influence of cannabidiol (CBD, 1 µM) on Nrf2 (**A**) and NFκB (p52) (**B**) nuclear localization in keratinocytes (control and exposed to UVA (30 J/cm^2^) and UVB (60 mJ/cm^2^) radiation). Nuclei were stained with Hoechst 33342 (blue) and antibodies against Nrf2 or NFκB (p52) were detected with fluorescent (FITC) secondary antibody (red dots).

Moreover, observed changes in proteins levels were accompanied with changes in translocation of transcriptional factors (Nrf2 and NFκB) to the nucleus. CBD led to the Nrf2 nuclear translocation in non-irradiated cells (Figure 7). However, the UVA induced Nrf2 nuclear translocation was slightly reduced by CBD, which was observed as increased Nrf2 level mainly in the cytosol. This effect was not observed in the case of cells treated with UVB and CBD. Different results were observed in the case of NFκB, where CBD treatment slightly enhanced NFκB levels in the cytoplasm but did not lead to its nuclear translocation. In the case of keratinocytes exposed to UV radiation the significant increase in NFκB level in the nucleus was observed, however, CBD treatment following UV radiation enhanced NFκB level in whole cells, but, especially in the case of UVA, prevented its accumulation in the nucleus.

## 4. Discussion

UVA and UVB radiation show different biological effects on skin cells, but the common characteristic of their action is to cause a redox imbalance, with a shift towards oxidation reactions [4]. This situation leads to the formation of oxidative stress as a result of which the metabolism of membrane phospholipids is increased and causes intensified generation of lipid mediators, which are products of reactions dependent on ROS or enzymes [43,44]. The main group of compounds generated during the enzymatic metabolism of phospholipids are endocannabinoids, which indirectly, by activating cannabinoid receptors (CB1/2), may modify the redox balance and inflammation in the cells, including UV irradiated cells [4,45,46]. Therefore, cannabinoids appear to be very promising therapeutic compounds.

One of the naturally occurring phytocannabinoids, and therefore a compound with potential protective effects, is CBD, which has antioxidant and anti-inflammatory properties [11,12]. It was demonstrated that this cannabinoid is a stronger antioxidant than α-tocopherol and ascorbic acid [47]. It has been also shown that in various diseases CBD reduces the intensity of ROS generation and oxidative stress formation, which decreases lipid mediators [48,49].

The results of this study indicate that CBD used under physiological conditions causes slight increases in the level of proinflammatory factors in the NFκB pathway and partially modifies the cytoprotective activity of Nrf2 in keratinocytes. This effect is not visible in cells with UV-induced metabolic stress, in which CBD partially normalizes redox balance and reduces inflammation in keratinocytes exposed to UVA and UVB radiation, which experience oxidative stress resulting from excessive production of ROS and consequently enhanced lipid peroxidation with 4-HNE as a main reactive electrophilic aldehyde as well as reduced antioxidant capacity, which reveals cytoprotective CBD (anti-inflammatory and antioxidant) properties that may indicate the need to adapt cells to pathophysiological conditions [50].

However, a completely different response of GSH- and thioredoxin-dependent antioxidant systems to CBD is observed for both unexposed and irradiated cells. The thioredoxin system consists of thioredoxin and thioredoxin reductase, which contains in its active site a highly reactive selenocysteine that is susceptible to oxidative (mainly by ROS) or electrophilic (mainly by 4-HNE) attack [51,52]. This makes it an important sensor of the redox cell state. Adducts created by 4-HNE can influence proliferation, differentiation and even cells apoptosis [53]. Therefore, in keratinocytes after CBD treatment, when ROS and lipid mediators levels are reduced, thioredoxin level and thioredoxin reductase activity are enhanced. Moreover, the data suggest that the thioredoxin system might reduce the oxidized cysteine residues in Keap1, a cytosol inhibitor of Nrf2 (the transcription factor responsible for cytoprotective protein biosynthesis), to restore its functionality [54]. On the other hand, in the case of the glutathione system, CBD decreases both the level of GSH and the activity of glutathione peroxidase. This may indicate a greater affinity of CBD for probably selenocysteine residues of these elements of the glutathione system. Moreover, CBD enhancing Nrf2 transcriptional activity leads to increase in the level of thioredoxin and thioredoxin reductase that levels are regulated by transcriptional action of Nrf2 [51]. Therefore, it is believed that the thioredoxin system is essential for the survival of keratinocytes, their protection against UV radiation and the improvement of wound healing [55]. However, this protection is not seen in CBD-treated keratinocytes because, as our results indicate, CBD modifies the structure of two of five essential cysteines in Keap1, leading to a decrease in its biological activity. Another explanation for this phenomenon may be the greater affinity of 4-HNE for glutathione than the thioredoxin system components and the interaction with the nucleophilic elements of this active/catalytic centers [56]. That may be confirmed by the decreased level of free 4-HNE in keratinocytes after CBD treatment.

These data show that CBD effectively supports the thioredoxin system, which is mainly responsible for maintaining the redox balance in the cytosol, while decreasing the efficiency of the GSH-dependent system, which is responsible for maintaining the redox balance in the mitochondrium and the endoplasmic reticulum [57]. Regardless of its direct antioxidant activity, cytoplasmic Trx regulates the activity of transcription factors, either directly (NFκB), or indirectly, i.e., AP-1, through interaction with the Ref-1 nuclear factor [58]. It demonstrates enhanced expression after UV radiation but reduced levels following CBD treatment. Under the influence of oxidative stress, both Trx and NFκB migrate from the cytoplasm to the nucleus, where Trx reduces the NFκB cysteine needed for its transcriptional activity [59]. Therefore, the CBD-induced increase in Trx level may protect against NFκB enhanced expression. Another equally important function of the thioredoxin-dependent system is participation in the regulation of gene expression by affecting apoptosis-regulating kinase (ASK-1) [60], whose expression is inhibited by a system of thioredoxins in UV-irradiated and CBD treated keratinocytes, thus protecting the cells from apoptosis, which may be the result of oxidative stress caused by radiation.

Increased expression of NFκB after UV irradiation and CBD treatment promotes enhanced expression of Nrf2, which induces the transcription of cytoprotective proteins required for resistance to stress [61]. However, Nrf2 activity depends on various activators and inhibitors [4]. Nrf2 is physiologically complexed with Keap1, causing this transcription factor to be the target for proteasomal degradation [62]. We showed that CBD modifies Keap1 Cys^151^ that participates in the Cul3-Keap1 complex formation that is needed for interaction with Nrf2. Because of decreased expression and structural modification of Keap1 in irradiated as well as CBD-treated keratinocytes, Nrf2 is transferred to the nucleus to a greater extent than in the control. Moreover, CBD increases the levels of Nrf2 activators, such as p21, KAP1 and p62 in control keratinocytes, while p21 is also increased in UV irradiated cells. In a non-canonical pathway, p62 activates Nrf2 in an autophagy-dependent manner, while Nrf2 positively regulates the expression of p62 genes and over expression of p62 is sufficient to inactivate Keap1 [63]. In addition, CBD tended to reduce the expression of PGAM5 phosphatase, which can interact with both Keap1 and Nrf2 [64]. At the same time, the expression of DPP3, a Keap1 activator, is increased. The combined effect of CBD on activators and inhibitors results in a significant reduction in Keap1 biological activity, which is seen as the enhanced expression of Nrf2.

As a consequence, increased Nrf2 target gene expression supports the stress resistance of irradiated cells and induces changes in metabolic pathways that participate in cellular defense by enhancing the removal of cytotoxic ROS and electrophiles including 4-HNE [65]. One of the classic activities of Nrf2 is the regulation of the HO-1 gene. It was earlier shown that HO-1 and its metabolites, such as CO and bilirubin, have significant anti-inflammatory activity mediated by Nrf2 [66]. However, HO-1 expression after UV radiation and CBD treatment is smaller than it can be expected after Nrf2 enhanced level. The results of this study confirm that the increased expression of Nrf2 and HO-1 after keratinocyte treatment with CBD results in a decrease in ROS generation, which in turn may inhibit the activation of the NLRP3 inflammasome. This mediates the secretion of the pro-inflammatory cytokine IL-1B, which induces cell death [67]. We have shown that CBD activates Nrf2 and partially inhibits UV-induced activation of NLRP3 and NFκB. However, the enhanced level of Trx as a result of CBD treatment could additional decrease the activation of the NLRP3 inflammasome. Therefore, the observed expression of the NLRP3 inflammasome is probably a result of CBD and Trx effects, which ultimately results in the inhibition of the expression of NLRP3. It has been previously shown that other natural and synthetic compounds (EGCG, biochanin) also demonstrate an inhibitory effect of Nrf2 on the activation of the NLRP3 inflammasome [68].

CBD under physiological conditions may stimulate the generation of many signaling factors- including the anti-inflammatory prostaglandin 15d-PGJ2, which can regulate COX activity. 15d-PGJ2 inhibits COX2 activity during inflammation but increases it under physiological conditions [69]. It was earlier shown that CBD reduces the activity of the NFκB pathway with a decrease in TNFα level [70]. Moreover, 15d-PGJ2 as an electrophilic molecule may form adducts with proteins, changing their structure and functions. This triggers important biological responses, including protection against inflammation as well as oxidative stress. 15dPGJ_2_ may exert anti-inflammatory activity by stimulating the Nrf2 pathway through the formation adducts with Keap1 and facilitate the dissociation of Nrf2 from the Keap1 adduct and consequent translocation of the transcription factor into the nucleus. This promotes up-regulation of cytoprotective genes, including HO-1 [71], which is also observed in this study after CBD treatment of UV irradiated keratinocytes. A similar effect may be demonstrated by interaction of electrophilic 4-HNE and PGJ_2_ with Keap1 [43,72].

On the other hand, oxidative stress and inflammation are believed to cause Nrf2 overexpression to protect cells from the effects of ROS overproduction as well as proinflammatory cytokines [73]. Therefore, if CBD is used, the observed overexpression of Nrf2 may be the result of the overproduction of TNFα, while the expression of TNFα may be dependent on both NFκB and Nrf2 activity. Nrf2 and NFκB cellular signaling pathways interact to control the transcription and functions of different proteins and it have been shown that direct or indirect activation and inhibition occur between members of the Nrf2 and NFκB pathways [39]. At the same time, suggested the role of Keap1 not only in Nrf2 but also in NFκB inhibition by complex formation [74], as well as Keap1 structure modifications by CBD additionally explain the observed increase in the level of both Nrf2 and NFκB factors. These study indicate that in UV and CBD treated cells, Nrf2 activation partially leads to inhibition the NFκB signaling pathway.

The transcription factor NFκB is in equilibrium with its inhibitor IκB, which is degraded in response to stress stimuli, such as after UV irradiation, and its expression increases after treatment with CBD. One of the inhibitors of NFκB-IκB complex is 4-HNE, which level increases under stress conditions but partially is decreased by CBD [43]. In addition, IκB activated kinase (IKK) results in the phosphorylation of NFκB, which promotes its release and translocation into the nucleus and transcription of pro-inflammatory mediators such as IL-6, TNF-α, iNOS and IL-1 [75]. It is also known that the Nrf2 inhibitor complex, Keap1/Cul3, may target the IKKβ element of IKK for ubiquitination and degradation [76]. A modified Keap1 level by CBD treatment indicated by our study and structure by 4-HNE and 15d-PGJ_2_ as was shown in the literature [43,77] due to UV and CBD treatment reduces the possibility of forming the Nrf2-Keap1/CuI3 complex and increases the transcriptional activity of both Nrf2 and NFκB. On the other hand, the increased expression of NFκB p65/RelA subunit after UV radiation and CBD treatment simultaneously imports Keap1 into the nucleus, where it terminates Nrf2 transcription of the genes by exporting this factor back to the cytoplasm [78]. However, when both transcription factors reach the nucleus, they need CREB binding protein (CBP) coactivators that are necessary for gene transcription [79], but the expression of CBP is already significantly increased by UV radiation and additionally by CBD treatment, so it probably does not require activators. Nevertheless, due to the action of UV and CBD, these two pathways can also be activated mutually, oxidative stress induced by NFκB activation facilitates Nrf2 translocation into the nucleus to protect cells against inflammation and oxidative damage.

## 5. Conclusions

These findings suggest that CBD activates Nrf2 and partially inhibits activation of NFκB. This activity is supported by cellular activators and inhibitors including lipid mediators such as 4-HNE and 15d-PGJ_2_, whose elevated level is a response to metabolic stress accompanying both UV irradiation and CBD treatment and which may interact with both Nrf2 and NFκB pathways.

## Figures and Tables

**Table 1:** The influence of cannabidiol (CBD) on ROS generation (by ESR) and antioxidant capacity estimated as protein (Cu,Zn-SOD, GSH-Px, GSSG-R, TrxR by spectrometry) and non-protein antioxidants (GSH by CE, Trx by Elisa, Ref-1 and phospho-ASK-1 by Western blotting) as well as lipid peroxidation (4-HNE by GCMS) of keratinocytes exposed to UVA (30 J/cm^2^) and UVB (60 mJ/cm^2^) irradiation.

Determined Parameter	Groups					
	Control	CBD	UVA	UVA + CBD	UVB	UVB + CBD
Superoxide anion (nmol/min/mg protein)	90 ± 7	58 ± 5 ^x^	240 ± 12 ^x^	87 ± 9 ^a^	216 ± 11 ^x^	105 ± 9 ^x,a^
Cu.Zn-SOD [mU/mg protein]	28.0 ± 1.5	48.4 ± 3.1 ^x^	23.1 ± 1.4 ^x^	51.4 ± 2.9 ^x,a^	21.5 ± 1.3 ^x^	53.6 ± 3.1 ^x,a^
GSH-Px [U/mg protein]	14.6 ± 0.9	13.5 ± 1.2	34.4 ± 1.9 ^x^	22.8 ± 1.7 ^x,a^	37.5 ± 2.2 ^x^	26.5 ± 1.5 ^x,a^
GSSG-R [mU/mg protein]	14.3 ± 1.2	7.2 ± 0.4 ^x^	18.6 ± 0.9 ^x^	26.5 ± 1.8 ^x,a^	20.2 ± 1.0	14.7 ± 0.9
GSH [nmol/mg protein]	17.1 ± 0.8	11.7 ± 0.7 ^x^	11.5 ± 0.6 ^x^	10.3 ± 0.6 ^x,a^	10.9 ± 0.5 ^x^	9.3 ± 0.5 ^x,a^
TxrR [U/mg protein]	14.0 ± 0.7	17.2 ± 0.8 ^x^	10.2 ± 0.6 ^x^	16.1 ± 0.7 ^x,a^	8.2 ± 0.3 ^x^	15.4 ± 0.6 ^x,a^
Trx [g/mg protein]	1.63 ± 0.09	2.74 ± 0.17 ^x^	1.05 ± 0.05 ^x^	1.79 ± 0.11 ^x,a^	0.84 ± 0.04 ^x^	1.16 ± 0.06 ^x,a^
Ref-1 [vs. control]	1.00 ± 0.06	0.96 ± 0.04	3.10 ± 0.07 ^x^	1.60 ± 0.06 ^x,a^	3.10 ± 0.09 ^x^	1.70 ± 0.06 ^x,a^
phospho-ASK-1 [vs. control]	1.00 ± 0.04	1.10 ± 0.04	2.10 ± 0.07 ^x^	1.70 ± 0.06 ^x,a^	2.30 ± 0.09 ^x^	1.60 ± 0.06 ^x,a^
4-HNE [nmol/mg protein]	26.1 ± 1.3	23.5 ± 1.4	52.4 ± 2.7 ^x^	35.6 ± 1.9 ^x,a^	73.4 ± 3.7 ^x^	64.7 ± 3.5 ^x,a^

Mean values ± SD of five independent experiments are presented. ^x^ statistically significant differences vs. control group, *p* < 0.05; ^a^ statistically significant differences vs. UVA or UVB group, *p* < 0.05.

**Table 2:** Effect of cannabidiol (CBD) on Keap1 expression and the level of its activators (determined by Western blotting) and inhibitors (determined by Western blotting–PGAM5 and by LCMS–4-HNE and 15dPGJ_2_) in keratinocytes exposed to UVA (30 J/cm^2^) and UVB (60 mJ/cm^2^) irradiation.

Determined Parameter	Groups					
	Control	CBD	UVA	UVA + CBD	UVB	UVB + CBD
Keap1 [vs. control]	1.00 ± 0.05	0.79 ± 0.04 ^x^	0.81 ± 0.04 ^x^	0.63 ± 0.03 ^x,a^	0.78 ± 0.04 ^x^	0.47 ± 0.02 ^x,a^
Inhibitors						
4-HNE [nmol/mg protein]	26.1 ± 1.3	23.5 ± 1.4	52.4 ± 2.7 ^x^	35.6 ± 1.9 ^x,a^	73.4 ± 3.7 ^x^	64.7 ± 3.5 ^x,a^
15d-PGJ_2_ [pg/mg protein]	6.2 ± 0.36	11.8 ± 0.86 ^x^	13.8 ± 0.81 ^x^	10.1 ± 0.60 ^x,a^	15.4 ± 0.91 ^x^	11.2 ± 0.72 ^x,a^
PGAM5 [vs. control]	1.00 ± 0.04	0.93 ± 0.05	0.88 ± 0.04 ^x^	0.81 ± 0.04	0.39 ± 0.02 ^x^	0.30 ± 0.02 ^x,a^
Activators						
WTX [vs. control]	1.00 ± 0.04	0.92 ± 0.05	0.82 ± 0.04 ^x^	0.75 ± 0.04 ^x^	0.75 ± 0.04 ^x^	0.45 ± 0.02 ^x,a^
DPP3 [vs. control]	1.00 ± 0.05	1.81 ± 0.09 ^x^	0.66 ± 0.03 ^x^	1.47 ± 0.07 ^x,a^	0.44 ± 0.02 ^x^	1.83 ± 0.11 ^x,a^

Mean values ± SD of five independent experiments are presented. ^x^ statistically significant differences vs. control group, *p* < 0.05; ^a^ statistically significant differences vs. group without cannabidiol, *p* < 0.05.
